# Supplementary material for: Modeling glioblastoma heterogeneity as a dynamic network of cell states
Source: Mol Syst Biol. 2021 Sep 16;17(9):e10105. doi: 10.15252/msb.202010105 (PMC8444284; doi:10.15252/msb.202010105)
Supplement: Supplementary file 6 — Source Data for Figure 5 [file MSB-17-e10105-s004.zip › Figure5A_sourcedata/GSEA_3071/nosigdb_stateI.GseaPreranked.1622795145664/GANDAL_IBD_UP_PMID29439242.html]

Details for gene set GANDAL\_IBD\_UP\_PMID29439242[GSEA]

|  || Dataset | state13071 |
| Phenotype | NoPhenotypeAvailable |
| Upregulated in class | na\_pos |
| GeneSet | GANDAL\_IBD\_UP\_PMID29439242 |
| Enrichment Score (ES) | 0.6304453 |
| Normalized Enrichment Score (NES) | 3.2916071 |
| Nominal p-value | 0.0 |
| FDR q-value | 0.0 |
| FWER p-Value | 0.0 |
Table: GSEA Results Summary

  

Fig 1: Enrichment plot: GANDAL\_IBD\_UP\_PMID29439242      
 Profile of the Running ES Score & Positions of GeneSet Members on the Rank Ordered List

  

| PROBE | GENE SYMBOL | GENE\_TITLE | RANK IN GENE LIST | RANK METRIC SCORE | RUNNING ES | CORE ENRICHMENT || 1 | MGP |  |  | 1 | 2.652 | 0.0565 | Yes |
| 2 | LUM |  |  | 4 | 2.203 | 0.1031 | Yes |
| 3 | DCN |  |  | 5 | 2.010 | 0.1462 | Yes |
| 4 | COL6A3 |  |  | 13 | 1.717 | 0.1809 | Yes |
| 5 | TGFBI |  |  | 17 | 1.674 | 0.2159 | Yes |
| 6 | KRT17 |  |  | 20 | 1.593 | 0.2494 | Yes |
| 7 | RARRES3 |  |  | 22 | 1.575 | 0.2829 | Yes |
| 8 | BGN |  |  | 24 | 1.568 | 0.3162 | Yes |
| 9 | COL4A1 |  |  | 28 | 1.524 | 0.3479 | Yes |
| 10 | IGFBP7 |  |  | 45 | 1.387 | 0.3730 | Yes |
| 11 | TIMP1 |  |  | 47 | 1.381 | 0.4023 | Yes |
| 12 | CTHRC1 |  |  | 49 | 1.370 | 0.4313 | Yes |
| 13 | CDH11 |  |  | 59 | 1.296 | 0.4565 | Yes |
| 14 | THY1 |  |  | 94 | 1.114 | 0.4705 | Yes |
| 15 | GPX8 |  |  | 133 | 0.959 | 0.4801 | Yes |
| 16 | KDELR3 |  |  | 136 | 0.951 | 0.4998 | Yes |
| 17 | WARS |  |  | 164 | 0.885 | 0.5110 | Yes |
| 18 | SAMD9L |  |  | 180 | 0.857 | 0.5250 | Yes |
| 19 | IGFBP5 |  |  | 198 | 0.833 | 0.5379 | Yes |
| 20 | XBP1 |  |  | 215 | 0.797 | 0.5504 | Yes |
| 21 | FKBP11 |  |  | 217 | 0.792 | 0.5670 | Yes |
| 22 | COL7A1 |  |  | 223 | 0.779 | 0.5823 | Yes |
| 23 | ALPL |  |  | 245 | 0.740 | 0.5921 | Yes |
| 24 | GBP1 |  |  | 248 | 0.736 | 0.6072 | Yes |
| 25 | IFITM3 |  |  | 268 | 0.714 | 0.6170 | Yes |
| 26 | S100A11 |  |  | 275 | 0.707 | 0.6304 | Yes |
| 27 | TRIM22 |  |  | 348 | 0.630 | 0.6231 | No |
| 28 | KLF2 |  |  | 428 | 0.571 | 0.6125 | No |
| 29 | LY96 |  |  | 451 | 0.547 | 0.6178 | No |
| 30 | OLFM4 |  |  | 487 | 0.524 | 0.6189 | No |
| 31 | ME1 |  |  | 543 | 0.484 | 0.6134 | No |
| 32 | EGR1 |  |  | 652 | 0.432 | 0.5914 | No |
| 33 | BHLHE40 |  |  | 720 | 0.405 | 0.5807 | No |
| 34 | CD55 |  |  | 726 | 0.403 | 0.5878 | No |
| 35 | GJA1 |  |  | 727 | 0.403 | 0.5965 | No |
| 36 | FOS |  |  | 832 | 0.370 | 0.5743 | No |
| 37 | IFITM2 |  |  | 836 | 0.370 | 0.5814 | No |
| 38 | FOSB |  |  | 854 | 0.364 | 0.5842 | No |
| 39 | ZC3H12A |  |  | 872 | 0.358 | 0.5870 | No |
| 40 | CASP1 |  |  | 981 | 0.331 | 0.5628 | No |
| 41 | IFI16 |  |  | 1062 | 0.315 | 0.5464 | No |
| 42 | SLC7A5 |  |  | 1084 | 0.310 | 0.5470 | No |
| 43 | ARFGAP3 |  |  | 1112 | 0.304 | 0.5457 | No |
| 44 | STAT1 |  |  | 1136 | 0.301 | 0.5454 | No |
| 45 | PHLDA1 |  |  | 1262 | 0.275 | 0.5152 | No |
| 46 | GZMB |  |  | 1273 | 0.274 | 0.5181 | No |
| 47 | CYR61 |  |  | 1318 | 0.265 | 0.5111 | No |
| 48 | GLIPR1 |  |  | 1346 | 0.260 | 0.5088 | No |
| 49 | UBE2L6 |  |  | 1370 | 0.256 | 0.5077 | No |
| 50 | TGM2 |  |  | 1444 | -0.252 | 0.4920 | No |
| 51 | PDE4B |  |  | 2132 | -0.339 | 0.3004 | No |
| 52 | PCDH17 |  |  | 2600 | -0.439 | 0.1747 | No |
| 53 | IL7R |  |  | 2665 | -0.460 | 0.1661 | No |
| 54 | CXCR4 |  |  | 3217 | -0.803 | 0.0238 | No |
| 55 | TNC |  |  | 3350 | -1.043 | 0.0080 | No |
| 56 | PDPN |  |  | 3472 | -1.788 | 0.0113 | No |
Table: GSEA details [plain text format]

  

Fig 2: GANDAL\_IBD\_UP\_PMID29439242: Random ES distribution      
 Gene set null distribution of ES for **GANDAL\_IBD\_UP\_PMID29439242**
